# Cancer burden, its pattern and survival in Muzaffarpur: findings from first population-based cancer registry of Bihar state, India

**DOI:** 10.3332/ecancer.2025.1972

**Published:** 2025-08-22

**Authors:** Atul Budukh, Sonali Bagal, Deepak Gupta, Sharyu Mhamane, Ravikant Singh, Burhanuddin Qayyumi, Abha Rani Sinha, Sanjay Kumar Singh, Satyajit Pradhan, Pankaj Chaturvedi, Rajendra Badwe, Sudeep Gupta

**Affiliations:** 1 Centre for Cancer Epidemiology (CCE-ACTREC), Tata Memorial Centre, Mumbai 410210, Maharashtra, India; 2 Homi Bhabha National Institute (HBNI), Training School Complex, Anushakti Nagar, Mumbai 400094, India; 3 Homi Bhabha Cancer Hospital and Research Centre (HBCH&RC), Muzaffarpur 842004, Bihar, India; 4 Sri Krishna Medical College and Hospital (SKMCH), Muzaffarpur 842004, Bihar, India; 5 Health Department, Government of Bihar, Patna 800015, India; 6 Mahamana Pandit Madan Mohan Malviya Cancer Centre (MPMMCC), Varanasi 221005, Uttar Pradesh, India; 7 Advanced Centre for Treatment Research and Education in Cancer (ACTREC), Navi Mumbai 410210, Maharashtra, India; 8 Tata Memorial Centre (TMC), Mumbai 400012, Maharashtra, India; a https://orcid.org/0000-0001-6723-802X; b https://orcid.org/0000-0002-2510-1751; c https://orcid.org/0009-0002-6101-7108; d https://orcid.org/0000-0002-7406-8134; e https://orcid.org/0000-0003-1829-3537; f https://orcid.org/0000-0002-3520-1342; g https://orcid.org/0000-0002-0480-2831; h https://orcid.org/0000-0002-6742-6378

**Keywords:** cancer control, cancer prevention, India, population-based cancer registry, rural and urban, cancer survival, incidence

## Abstract

**Background:**

The first population-based cancer registries (PBCRs) in Bihar state, India was established at Muzaffarpur by the Tata Memorial Centre (TMC), Mumbai. This article presents the cancer burden, its pattern for the years 2018-2021 and population-based survival for the years 2018 cases followed till 2023.

**Methods:**

The registry follows an active method of case finding which includes visits to the hospital, diagnostic and treatment facilities centres, birth and death registration office. Cases were collected through village visit, community interaction and verbal autopsy. After quality and consistency checks by senior staff of TMC, Mumbai; data are entered into the CanReg5 software. The cancer registry has faced several challenges in data collection, such as poor maintenance of medical records noncooperation of the hospital and patient’s relatives reluctant to share the cancer case information. Most patients travel long distances for diagnosis and treatment. The challenges faced by the registry were overcome with the help of the administrative support of the district authorities.

The rates were calculated using standard registry methods. The survival of 2018 incidence cases (followed till 31st December 2023) was calculated by using the Kaplan-Meier and Pohar Perme method.

**Results:**

In the period 2018–2021, a total of 2,916 cancer cases (Male: 1,436 (49.2%) and Female: 1,480 (50.7%)) were registered. The incidence rates for males and females were 40.2 and 46.8 per 100,000 population, respectively. Whereas 2,076 cancer deaths (Male: 1,049 (50.5%) and Female: 1,027 (49.5%)) were registered and mortality rates were 29.6 and 32.6 per 100,000 for males and females, respectively. The leading cancer sites for males are mouth (AAR 6.0), tongue (2.6), prostate (2.0), gallbladder (1.9), liver (1.6); and for females, breast (11.1), cervix uteri (6.3), gallbladder (5.2), lung (1.9) and ovary (1.6).

Among men, 5-year age-standardised relative survival (age 0–74 years) of mouth, prostate and tongue cancer cases were 25.59%, 30.41% and 31.90%, respectively. Similarly, among females, it was 32.39% of breast, 20.73% of cervix uteri. None of the gallbladder cases survived after 3 year and 5 years of diagnosis.

**Conclusion:**

The population-based cancer registry has successfully generated good-quality data, which can be utilised to plan cancer control programs, enhance the infrastructure for cancer care and facilitate etiological research in this population. Given the poor survival of leading sites in Muzaffarpur, emphasis must be laid on strengthening effective cancer control strategies for these cancers.

Due to several challenges faced by the registry, we have noted underreporting. In the coming years, due to improvements in the infrastructure and raising awareness about the use of registry data in planning cancer care services, we are expecting an improvement in cancer registration.

## Introduction

Bihar is the third most populous state in India, with a population of 104 million as per the 2011 census. It is predominantly rural (88.7%). Bihar has 38 administrative districts, of which Muzaffarpur district forms the northern part of Bihar [1, 2]. Bihar ranks the lowest in terms of literacy rate; there also exists a lack of cancer awareness among the population. As per the National Family Health Survey 5, less than 1% underwent cancer screening for cervical, breast and oral cancers, respectively, in Bihar state [3]. Due to low awareness about the disease and limited cancer care infrastructure, there is a lack of access to diagnosis and treatment. Therefore, most of the cancer cases reach the hospital at an advanced stage of disease.

As per GLOBOCAN 2022, in India by 2050; incident cancer cases are estimated to rise by 90.4% and mortality by 99% [4]. This estimation calls for revamping cancer control measures in the country. The cancer registry serves as a mirror to reflect on the cancer burden in any given geographic area and is an essential component of cancer control strategies. As of 2024, the network of cancer registries in India consists of 52 PBCRs, of which, only a few are rural cancer registries [5].

Bihar had no PBCR data before 2018. Establishing a PBCR in Bihar was quite challenging as a majority of the population resided in the rural areas while the cancer treatment facilities were concentrated in the urban area of Patna; the capital city of Bihar. Due to inaccessible diagnostic and treatment facilities, there were issues related to advanced/late-stage diagnosis and lack of treatment compliance. Despite these hurdles, Tata Memorial Centre (TMC), Mumbai, India established the Muzaffarpur PBCR in the year 2018, which was the first cancer registry to be established in Bihar. The registry is functioning with the help of Sri Krishna Medical College and Hospital, Muzaffarpur and Health Department, Government of Bihar and released its first report in 2023 [6]. Through this paper, we aim to present the cancer incidence and mortality for the year 2018–2021 and survival of the cancer cases registered in 2018 in the Muzaffarpur (Bihar) using PBCR Muzaffarpur data.

## Methodology

### Registry area and population covered

**Figure 1.**
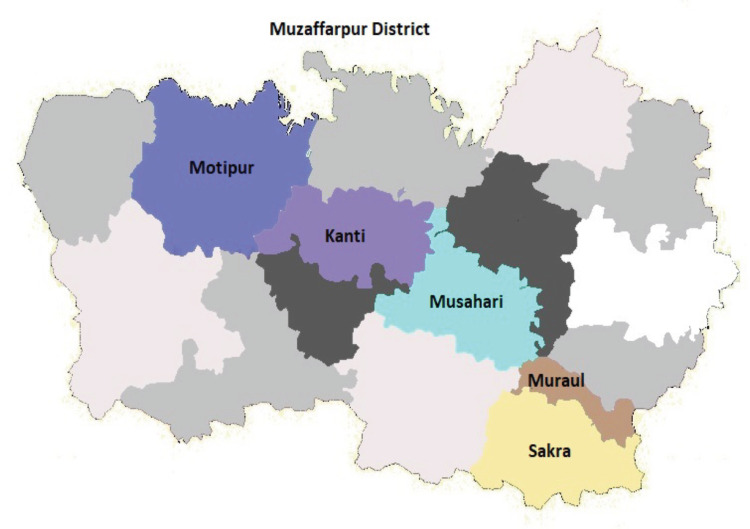
Registry area covered by the Muzaffarpur population-based cancer registry.

The Muzaffarpur PBCR covers five blocks of the district namely: Kanti, Motipur (Baruraj), Muraul (Dholi), Musahari, Sakra and Muzaffarpur Municipal Corporation, with an estimated population for the year 2018 of 2 million. Though there are several blocks in the Muzaffarpur district, due to funding limitations and lack of human resources, only the above-mentioned blocks were covered by the PBCR. The registry covers urban areas (83 wards) as well as 528 villages in the district. As per the 2018 estimated population of the registry area, 78.4% of the population covered by PBCR is rural. The registry area is presented in Figure 1.

### Staff recruitment and training

Field supervisor and staff were selected from the district and were trained in cancer registry operations at the Centre for Cancer Epidemiology (CCE-TMC), Mumbai. The staff were provided training on the role of cancer registry and its role in cancer control, cancer registration, case abstraction, International Classification of Diseases for Oncology – Third Edition, Geneva, 2000 (ICD-O3) coding, [7] data entry in CanReg5 software [8] and data analysis. For cancer registration field training, the staff was deputed to the well-established PBCRs of Varanasi and Sangrur in North India. Periodical refresher training programs were conducted by the CCE-TMC either in person or virtually.

### Cancer registration methodology

The PBCR follows the active method of case findings, wherein the registry staff visits the allotted villages every 6–8 months. They conduct group meetings with Auxiliary Nurse Midwives (ANMs), Anganwadi workers, Accredited Social Health Activists (ASHA) workers, JEEViKA workers (staff appointed by the Bihar Rural Livelihood Promotion Society) and key stakeholder to collect information on cancer cases and deaths. After interacting with the patients/relatives, the registry staff collects the details of the cancer cases and confirms them through respective sources. The source of cancer case information includes the village administration office, hospitals, medical colleges, cancer centres, radiology and pathology laboratories, as well as the birth and death registrar’s office. The registry staff visits these sources, collects relevant clinical details of the cancer case and registers the case in the prescribed proforma. In case of inaccessibility to the medical records of the patient, staff register the case based on the information provided by the patient/patient’s relatives. Muzaffarpur PBCR covers more than 50 sources, including the ones from Patna, Darbhanga, Varanasi and Mumbai [6].

In addition to source visits, the registry staff also interacts with the community leaders and the medical officer and staff of the Primary Health Centre. This helps them collect information regarding cancer cases diagnosed in the village as well as any cancer deaths that have occurred in the village. The community leaders play a pivotal role in cancer registries by helping in capturing the cancer cases, which is difficult to achieve with a wider geographic spread [9]. The interaction with patient relatives and community leaders ascertains the patient’s residence information.

During the subsequent visits to the village, the registry staff updates the vital status of the cancer cases. He/she also identifies any new cancer cases/deaths in the village. This is a continuous process of village visits.

### Data checking and quality control

All the cases collected by field staff from the villages as well as from the hospitals are scrutinised by the senior staff member at CCE-TMC. After residence confirmation (resident of the Muzaffarpur registry area for at least 1 year), and duplicate checking the cancer case information is documented in a prescribed format. The primary site and histology are coded based on the ICD-O3. Periodically, data has been sent to CCE-TMC for data quality control. All difficult cases have been discussed with clinicians and then coded appropriately [6].

### Data entry

**Figure 2.**
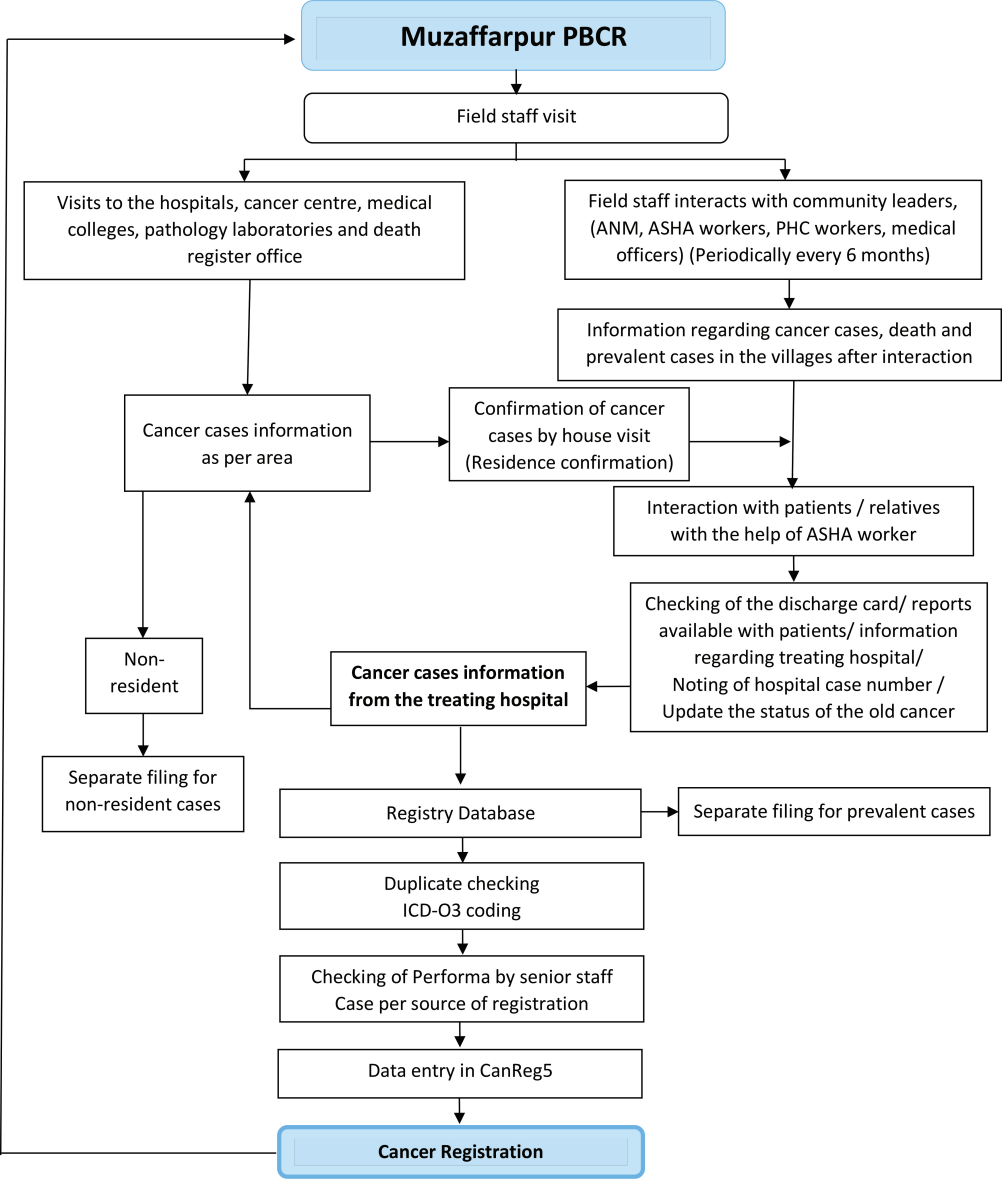
Registry methodology flowchart for the Muzaffarpur PBCR, Bihar, India.

Data were entered in CanReg5 software developed by the International Agency for Research on Cancer (IARC), Lyon, France [8]. After duplicate checking and other quality control measures, cancer incidence and death cases are registered. The registry methodology is illustrated in Figure 2.

### Challenges faced by Muzaffarpur Cancer registry

The Muzaffarpur Cancer Registry faces several hurdles in its operation. These include challenges in data collection due to non-participation from the private hospitals as well as the community, which led to the under-reporting/under-registration of the cancer cases. Due to a lack of awareness about the disease among the general population. It is most often that once a patient passes away, their family members burn their medical records. Relatives were reluctant to share the details of cancer patients with the registry staff and often expected some benefit in return for the information. In such cases, we have used the verbal autopsy technique to register the cancer case [10]. In addition, poor documentation of medical records and poor health infrastructure made it difficult for the staff to trace the primary site of the cases; therefore, cases had to be recorded as unknown primary [6]. The primary unknown cases were registered based on clinical details and verbal autopsy. For unknown primary cases registered based on clinical details: These include cases that were diagnosed in advanced stages, where metastasis was mentioned and the primary site was not confirmed. Such cases were classified as unknown primary. For cases registered through verbal autopsy: In cases where the patient’s relative was unable to mention/share the site of cancer, the case was classified as unknown primary. The higher number of unknown primary cases reflects the lack of diagnostic and treatment facilities in a given region [11]. This leads to heavy reliance on verbal autopsy while collecting cancer details from the patient and their relative.

Despite the hurdles, Muzaffarpur PBCR has managed to present reliable data that helps in understanding the cancer patterns in this region. Even in the period of the COVID-19 pandemic data collection was hampered.

### Statistical analysis

The population for 2018–2021 was estimated by the ratio method using 2001 and 2011 census populations [12]. Other measures such as the age-specific rates, age-adjusted rates (AAR), Truncated rates and ‘Lifetime risk’ were calculated using CanReg5 and SPSS software Version 21 (IBM, Armonk, New York, USA). The urban-rural rate ratio was calculated using the world standard population along with a 95% confidence interval [13].

For survival analysis of leading sites, we have considered the cancer cases diagnosed in the year 2018. The date of incidence was the starting date and the cases were followed up to 31st December 2023. The observed survival was calculated using the Kaplan-Meier method, while the relative survival was calculated using the Pohar Perme method [14]. Cancer cases lost to follow-up were excluded from the analysis.

## Results

In the period 2018–2021, a total of 2,916 cancer cases were recorded by the Muzaffarpur PBCR, consisting of 1,436 (49.2%) male and 1,480 (50.7%) female cancer cases. Of the total 2,916 cancer cases; 65.8% of cases were microscopically verified, 3.3% on a clinical basis, 7.2% on a radiological basis and verbal autopsy was (22.8%) and DCO (2%).

The age-adjusted incidence rate per 100,000 population for males was 40.2 and 46.8 for females. The cumulative risk in males aged 0–74 years was 4.4% (1 in 23 males were at risk of developing cancer) and in females, it was 5.1% (1 in 20 women were at risk of developing cancer).

**Figure 3.**
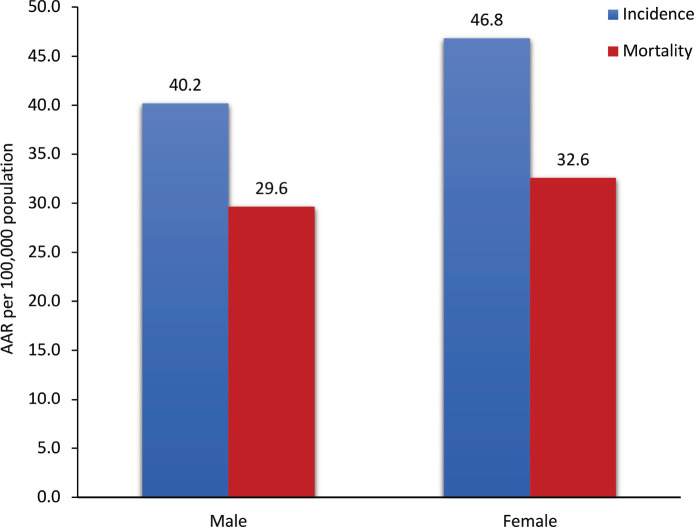
Cancer incidence and mortality age-adjusted rates (AARs) in Muzaffarpur area: 2018-2021.

A total of 2,076 cancer deaths were recorded for the years 2018–2021, of which 1,049 (50.5%) were male and 1,027 (49.5%) were female cancer deaths. The age-adjusted mortality rate was 29.6 and 32.6 per 100,000 population for males and females, respectively. The cumulative risk of death due to cancer in males aged 0–74 years was 3.2% (1 in 31 males were at risk of dying due to cancer) and in females, it was 3.6% (1 in 28 women were at risk of dying due to cancer). The cancer incidence and mortality rates in the Muzaffarpur area for the years 2018–2021 are presented in Figure 3. The overall mortality to incidence (M:I) ratio is 0.71 (male: 0.73, female: 0.69).

**Figure 4.**
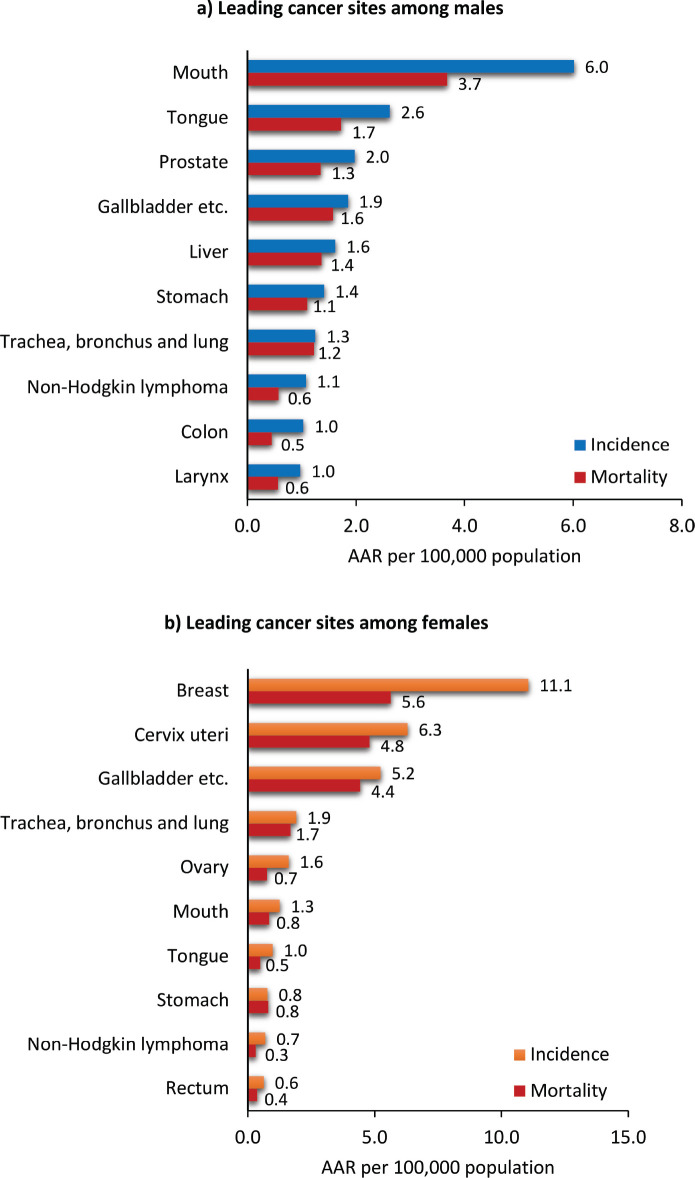
Leading cancer sites among males and females in the Muzaffarpur area: 2018-2021. AAR: Age adjusted rates

The leading cancer sites for males are the mouth (AAR 6.0 per 100,000 population), tongue (2.6), prostate (2.0), gallbladder (1.9) and liver (1.6). The leading cancer sites among females were breast (11.1 per 100,000 population), cervix uteri (6.3), gallbladder (5.2), trachea, bronchus and lung (1.9) and ovary (1.6). The leading cancer sites among males and females are presented in Figure 4. The comparison of the leading sites of Muzaffarpur with other cancer registries is shown in Table 1 [6, 15–22].

### Rural-urban disparity

The cancer incidence rates were different for the rural and urban areas. The overall incidence is 60% higher in the urban areas as compared to that of the rural region for males (RR:1.6, 95% CI (1.36–1.76)). A similar observation was noted for females, where the rates were 40% higher in urban than rural areas (RR:1.4, 95% CI (1.25–1.60)), the difference was statistically significant. Table 2 illustrates urban-rural differences by site and sex.

The age-adjusted incidence of mouth cancer among males is 2.5 times higher in the urban area (11.5) as compared to the rural (RR:2.5, 95% CI (1.79–3.59)), and the difference was statistically significant.

For tongue cancer incidence among males, AAR was 2.4 times higher in urban areas (4.9) as compared to rural areas (RR:2.4, 95% CI (1.44–4.03)). The difference between rates was statistically significant.

The incidence of gallbladder cancer among both males and females shows 30% higher AAR in urban (males: 2.3, females: 6.3) as compared to the rural area (males: 1.7, females: 4.9). However, the observed difference is not statistically significant among both males (RR:1.3, 95% CI (0.69–2.45)) and females (RR:1.3, 95% CI (0.88–1.87)).

The burden of breast cancer was observed to be more than two-fold among females in urban (19.0) as compared to rural (8.9) and the difference was statistically significant (RR:2.1, 95% CI (1.63–2.81)).

In contrast, the rates of cervix uteri cancer were slightly higher in the rural (6.4) as compared to urban (6.1); however, the difference was not statistically significant (RR:1.0, 95% CI (0.68–1.35)).

### Pediatric cancer

**Figure 5.**
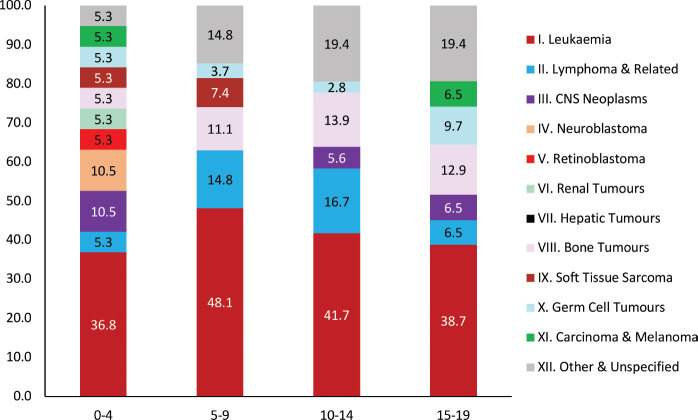
Proportion of pediatric cases by age group for both boys and girls (2018-2021).

Of the total cancer cases 82 (2.8%) [Boys: 55, Girls: 27] pediatric cases registered in the age group 0–14 years and 113 (3.9%) (Boys: 72, Girls: 41) in the age group 0–19 years. The pediatric age group ranges from 0 to 14 years of age. The cancer incidence AAR for boys in the 0–14 age group is 35.3 per million population and 17.7 per million for girls. The majority of cancer in the pediatric age group was Leukaemia, which consisted of more than 40% of the cases, followed by Lymphoma and related categories. The distribution of pediatric cancer is presented according to the format of International Classification for Childhood Cancer, Third Edition , [23] which presents the pediatric cancer burden for the age groups from 0 to 19 years of age. The site-wise distribution of pediatric cancer cases is presented in Figure 5.

### Tobacco-related cancer

Tobacco-related cancer (TRC) consists of cancer in those anatomical sites that are affected by the consumption of tobacco. As per IARC, WHO, Lyon, France, these sites include lip (C00), tongue (C01-C02), mouth (C03-C06), pharynx (C10, C12-C14), oesophagus (C15), larynx (C32), lung (C33-C34) and urinary bladder (C67) as these are associated with the use to tobacco [24].

The TRC rates in Muzaffarpur for the years 2018–2021 are 14.2 and 5.5 per 100,000 among males and females, respectively. The comparison of TRC rates of Muzaffarpur with neighbouring cancer registries is presented in Table 3 [6,15–22].

### Survival of leading cancers in Muzaffarpur for the year 2018

Based on the Muzaffarpur Cancer Registry report (2018), [6] it was observed that mouth, prostate and tongue were the leading cancer sites among males. In females, the breast was the leading cancer site, followed by the cervix uteri and gallbladder. These cancer cases were followed till 31st December 2023 for vital status. Survival of leading cancer sites was analysed based on the data of 298 patients. Out of 298 cancer cases, 104 (34.9%) were males and 194 (65.1%) were females. In 2023, out of 104 male cancer cases, 27 (26%) were alive and 77 (74%) died. However, among females, 39 (20%) cancer cases were alive, 153 (79%) died and 2 (1%) cases were lost to follow-up (excluded from the analysis).

**Figure 6.**
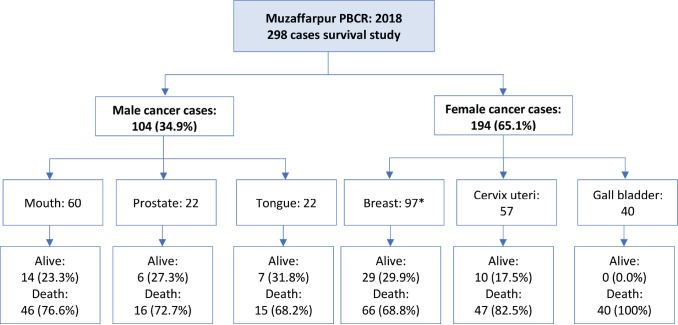
Classification of cases included for survival analysis.

The illustration of cases excluded from the survival analysis is presented in Figure 6.

As the registry staff follows an active method of case follow-up, we have the updated vital status for 298 cancer cases. Two cases whose vital status could not be followed up by registry staff were excluded from the analysis.

Among men, the 5-year relative survival rates of mouth, prostate and tongue cancer cases were 23.89% (95% CI: 13.99–35.26), 26.89% (95% CI: 10.72–46.19) and 31.46% (95% CI: 13.89–50.79), respectively. Similarly, among females, it was 32.75% (95% CI: 23.54–42.25) of breast, 21.56% (95% CI: 11.97–32.98) of cervix uteri and none of the gall bladder cases after 3 years were alive in females. It was observed that around 85% of the gallbladder cases died within 1 year of the diagnosis.

**Figure 7.**
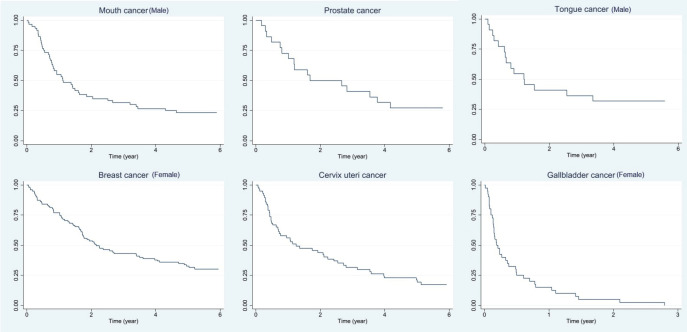
Overall survival of leading cancer sites among males and females in Muzaffarpur (2018).

The tabular illustration of the relative and observed survival for 1, 3 and 5 years is given in Table 4. The overall survival of leading cancer sites is presented in Figure 7.

The site-wise age-standardised relative survival (ASRS) rates of Muzaffarpur PBCR for the year 2018 are presented in Table 5. The 5 years ASRS for 0–74 age group among males is 25.59% for mouth (95%CI: 15.18–37.32), prostate 30.41% (95%CI: 12.68–50.36) and tongue 31.9% (95%CI: 14.72–50.59). While for females, it was 32.39% (95%CI: 23.12–41.98) for breast and 20.73% (95%CI: 11.12–32.39) for cervix uteri cases.

## Discussion

### Registry challenges and cancer burden

Muzaffarpur PBCR is the first PBCR established in the state of Bihar, India, which is predominantly rural. Several challenges are faced by the Muzaffarpur Cancer Registry. These include reluctance of sources to share the data regarding cancer patients due to data privacy concerns, lack of awareness of the cancer registry’s purpose, expectations of some benefits and compromised/poor quality of medical records. Patients travel around 60–2,000 km in the neighbouring states/districts for treatment; [6] thus, tracing these cases is difficult. Patients often opt for alternative forms of treatment, which do not have a clinical/medical note to verify the case. In addition to this, the COVID-19 pandemic hampered the registry activities. Staff sustainability is also an issue, wherein the trained staff quit the registry and retraining the new staff was time and resource-consuming. To add to the field-related challenges, in Bihar, there are natural calamities and unfavourable weather conditions, which further create hurdles for the registry staff to register cancer cases.

The initial period of the registry establishment was challenging and there was under-reporting/under-registration of the cases; there might also be chances of poor diagnostic labelling as the access to treatment was compromised. Similar observations were highlighted by the Sangrur, Varanasi, Kolkata and Dindigul, Ambilikkai cancer registries [21, 22, 25, 26]. Despite the obstacles, the registry staff overcame all these hurdles and reported good quality data on cancer cases in the Muzaffarpur area of Bihar, as the data were thoroughly and periodically scrutinised by the senior staff at TMC, Mumbai.

To overcome the challenges, help was sought from the Health Department of the Government of Bihar and the district health authorities of the Muzaffarpur district by issuing orders to government and non-government institutions to share the cancer patients’ data for registry activity. Trust among the community was acquired due to regular interactions of the field staff with the community leaders, ANMs, ASHA, JEEViKA workers and Anganwadi workers. Additionally, to minimise frequent visits to different cancer centres, the registry staff has requested these centres to share the data electronically.

This insight presents the cancer pattern in the Muzaffarpur area of Bihar for the years 2018–2021. The overall incidence and mortality rate in Muzaffarpur are lower than in any other Indian registry [27]. These findings are low as compared to the Varanasi cancer registry of the neighbouring state, as well as the registries of Kathmandu (Nepal) and Bhutan [18, 19]. However, it is the initial years of the registry may be due to under-reporting and further observations for 5 years will give a change in the cumulative risk of cancer among the population. The pattern of breast, cervix uteri and gallbladder cases is similar to the Varanasi registry [9].

### Pediatric cancer burden

The pediatric cancer cases in the Muzaffarpur area were noted to be extremely low. Research conducted to give a situational analysis of childhood cancer services noted that there is a lack of dedicated pediatric oncology units in all tertiary care hospitals. The burden of pediatric cancers in Muzaffarpur is lower than the national average. However, it is observed that the rates for girls are lower than the boys, which is consistent across all the India PBCRs [28–30]. This highlights the need for improvement in the diagnostic and treatment infrastructure for pediatric cancers in Bihar as well as other parts of India.

### Rural-Urban disparity

The cancer burden was disproportionately high in the urban areas as compared to the rural areas of Muzaffarpur and the difference was statistically significant. Similar findings were noted at the Varanasi and Punjab cancer registries, cancer burden was higher among the urban areas as compared to that of the rural [31, 32]. The leading cancer sites among males were mouth, tongue, prostate, gallbladder and liver. The leading cancer sites among females were breast, cervix uteri, gallbladder, trachea, bronchus lung and ovary. The urban-rural disparity highlights the lack of access to cancer diagnosis and treatment, besides other lifestyle factors. This gap hinders the identification of the true epidemiological burden in the rural area. Similar rural-urban disparities were observed in other Indian cancer registries [9, 31–33].

### Survival of common cancers in Muzaffarpur, Bihar

The active follow-up method for vital status is applied by the Cancer Registry. Due to the community involvement and interaction with patients and their relatives, the cancer registry was able to update the vital status of almost 99% of cancer patients; Sangrur PBCR also used a similar strategy [34]. Survival analysis based on 2018 data of 298 patients registered at the Muzaffarpur Cancer Registry. Among men, the 5-year age-standardised relative survival (0–74 years) of mouth, prostate and tongue cancer cases were 25.59%, 30.41% and 31.90%, respectively. Similarly, among females, it was 32.39% of breast, 20.73% of cervix uteri and none of the gall bladder cases after 3 years were alive in females. A population-based study reported a 5-year overall oral cancer survival of 32.3% for the years 2010–2016 [35]. In a recent oral cancer survival study based on 10 PBCRs from India showed the overall 5-year ASRS rate was 37.2%. In this study, Ahmedabad urban had the highest 5-year ASRS at 58.4% for both sexes, while Manipur had the lowest rate at 20.9% [36]. The survival rates of Manipur and Muzaffarpur were in a similar range. The 5-year age-standardised relative survival (0–74 years) of cervical cancer among women was lower in Muzaffarpur as compared to 35.7% among women in the Barshi rural cancer registry [37]. Similarly, studies using the 11 population-based cancer registries in India data show 5-year standardised relative cervical cancer survival was 51.7%; the highest age-standardised relative survival among these PBCRs was in Ahmedabad urban (61.5%), followed by Thiruvananthapuram (58.8%) [38]. A similar study for breast cancer was conducted and estimated by a 5-year age-standardised relative survival was 66.4% [39]. As per Surveillance, Epidemiology and End Results, the 5-year relative survival of breast cancer is around 91% and 67% for cervical cancer and oral cancer is above 60% for the period 2014–2020 [40]. The higher survival in developed countries can be attributed to better cancer control activities.

### Gallbladder cancer

Gallbladder cancer (GBC) is a common malignancy in Western Bihar and Eastern Uttar Pradesh, a highly lethal digestive tract cancer, majorly affecting women [41, 42]. A substantial rise in the incidence of GBC cases in Bihar, India has been seen [41]. GBC does not exhibit any symptoms in the early stages and is often diagnosed at the advanced stage of the disease (III or IV) and therefore regarded as the ‘silent killer’ of the Indo-Gangetic basin [42, 43]. In this paper, the survival reported for GBC is poor as none of the cases of GB survived for 3 years after diagnosis. The plausible risk factors for rising GBC are attributed to environmental, household and dietary factors. During field activity, health education on gallbladder cancer among the community and primary care among physicians must be provided.

### Oral, breast and cervical cancers

The other predominant cancers in Bihar include the cancers of the head and neck [44, 45]. This is due to the high consumption of tobacco in the region. A survey conducted in 0.1 million households in India found that Bihar had the highest prevalence of household consumption of smokeless tobacco (57%) as compared to the other Indian states [44] Khaini was the predominant smokeless tobacco of choice [46]. Our study compared the rate of tobacco-related cancers among the neighbouring states and found that the rate of tobacco consumption was high in Muzaffarpur and Varanasi as compared to other states. To curtail this longstanding problem of tobacco consumption in these areas, it is essential to lay special emphasis on prevention strategies such as school education, cancer screening camps and promoting the use of tobacco quitline services [46]. These strategies, when coupled together, will form a basis for establishing tobacco control activities in the region.

Breast cancer is the most common in Bihar after head neck and gallbladder cancer [47] Limited awareness, lack of effective cancer control activities, sociocultural barriers to seeking treatment and limited health infrastructure compel patients to travel long distances to seek treatment. The literature states long-distance travel for cancer-directed treatment was a hurdle for treatment completion [48–50].

Cervical cancer risk factors particular to Bihar include poor genital hygiene, lack of clean water and sanitation, and poor diet leads to poor immunity, which in turn results in persistent chronic infections such as human papillomavirus [51]. Further, girls are married early and usually have multiple pregnancies. Sexual exposure and poor genital hygiene during adolescence leads to increased risk of cervical zone infection. This leads to the initiation of malignancy. In addition to this, low literacy rates, limited trained oncologists and a deficit of cancer-directed treatment and infrastructure in Bihar lead to late-stage presentation and increased burden of cancer [1,52–54].

For oral, breast and cervical cancer, a model of cancer prevention by providing opportunistic cancer screening, health education and easy access to diagnosis and treatment was used in Sangrur [54]. A similar model can be replicated in Muzaffarpur, Bihar to raise awareness about the disease, provide easy access to diagnosis and treatment as well as improve survival.

In recent years, TMC has established cancer treatment facilities and has revamped the surgical and chemotherapy units. The radiotherapy services will be commenced soon. The expanding cancer treatment infrastructure in the Muzaffarpur district through Homi Bhabha Cancer Hospital and Research Centre at Muzaffarpur will improve the survival outcomes among the patients.

### Role of cancer registry in cancer control

Population-based cancer registry plays an important role in understanding the patterns of cancer in a given geographical area. The data generated from cancer registries form a basis for conducting several epidemiological studies [55]. Identifying patterns could be a precursor to confirming and identifying new risk factors peculiar to that region. This will in the future help in formulating effective prevention strategies and developing cancer care infrastructure to cater to the needs of the population. The cancer registry of Muzaffarpur will monitor the cancer burden and present timely data for the same. Before the establishment of the Muzaffarpur Cancer Registry, obtaining cancer data from this region was difficult [6, 44]. Through this paper, researchers as well as the oncologists can gain insight into the cancer profile of the Muzaffarpur area. Due to the initial period of registry establishment, the registry faced several challenges and, therefore, there was underreporting of the cases. This was majorly due to the refusal of data sharing from the private facilities as well as the community. Additionally, the COVID-19 pandemic further had an impact on the completeness of the data. However, as the registry progressed, these challenges were overcome with the help of the Health Department of the Government of Bihar and community interaction with the help of ASHA, ANM, Anganwadi and JEEViKA workers and by regular refresher training of the registry staff. Due to several challenges faced by the registry, we have noted underreporting. In the coming year, due to improvements in the infrastructure and raising awareness about the use of registry data in planning cancer care services, there will be improvement in the cancer registration.

## Conclusion

The present study provides a comprehensive overview of the cancer burden, its pattern and survival in the Muzaffarpur area, Bihar, through an analysis of population-based cancer registry data from 2018 to 2021. The findings highlight critical patterns in cancer incidence, types and demographic distributions, underscoring the region’s unique epidemiological profile.

The rising incidence of cancers, particularly those associated with lifestyle factors, environmental exposures and limited access to healthcare services, calls for urgent public health interventions. This study emphasises the importance of population-based cancer registries in identifying trends and facilitating data-driven policy formulation. It advocates for sustained efforts to strengthen cancer surveillance and integrate preventive measures, especially in under-resourced settings like Muzaffarpur, to improve health outcomes and reduce the disease burden. This will be achieved with a strengthened cancer registry that will also serve as a pathway to monitor cancer control activities in Bihar.

Since there is limited accessibility to cancer care facilities in Muzaffarpur, the government should work towards developing a proper referral system that connects the primary health centre to tertiary care. Patient navigators must be appointed to ease the process of obtaining cancer care, as they play an essential role in patient diagnosis and treatment compliance. Funding should be provided to strengthen the existing and expanded cancer care infrastructure in the state.

## List of abbreviations

AAR, Age-adjusted rates; ANM, Auxiliary Nurse Midwives; ASHA, Accredited Social Health Activist; ASR, Age-specific rates; CCE, Centre for Cancer Epidemiology; GBC, Gallbladder cancer; IARC, International Agency for Research on Cancer; ICD-O3, International Classification of Diseases for Oncology, Third Edition, PBCR, Population-based cancer registry; TMC, Tata Memorial Centre; TR, Truncated rates; TRC, Tobacco-related cancer.

## Conflicts of interest

The authors declare no conflicts of interest.

## Funding

The authors declare no conflicts of interest.

## Statement of ethical approval

This is a regular public health programme run with the help of the district health authorities of the state government.

## Author contributions

AB: Conceptualisation, methodology, writing, editing, critical reviewing and overall supervision;

SB: Data management, data analysis, quality control and assistance in writing the manuscript;

DG: Data curation, data analysis, data management and assistance in writing the manuscript;

SM: Writing and editing, literature review and visualisation;

RS: Meeting with government authorities, literature review and assistance in writing the manuscript;

BQ: Data quality control, literature review and assistance in writing the manuscript;

ARS: Critical reviewing and assistance in writing the manuscript;

SKS: Coordination with district health authorities and assistance in writing the manuscript;

SP: Critical reviewing and assistance in writing the manuscript;

PC: Critical reviewing and overall supervision;

RB: Critical reviewing and overall supervision;

SG: Critical reviewing and overall supervision.

## Figures and Tables

**Table 1.** Comparison of leading sites of Muzaffarpur PBCR with other Indian cancer registries with neighbouring and developed countries.

Cancer registry (Year)	Male (AAR* per 100,000 population)					Females (AAR* per 100,000 population)				
	Mouth	Tongue	Prostate	Gallbladder	Liver	Breast	Cervix Uteri	Gallbladder	Lung etc.	Ovary
Muzaffarpur, India (2018–2021)	6.0 (5.18–6.82)	2.6 (2.07–3.13)	2.0 (1.54–2.46)	1.9 (1.43–2.37)	1.6 (1.17–2.03)	11.1 (9.91–12.29)	6.3 (5.41–7.19)	5.2 (4.39–6.01)	1.9 (1.41–2.39)	1.6 (1.16–2.04)
Varanasi, India (2018–2019)	18.4 (16.95–19.85)	5.6 (4.80–6.40)	3.3 (2.61–3.99)	4.2 (3.49–4.91)	3.8 (3.12–4.48)	13.1 (11.86–14.34)	7.2 (6.26–8.14)	8.2 (7.19–9.21)	2.2 (1.67–2.73)	3.9 (3.23–4.57)
Sangrur, India (2017–2018)	3.8 (2.88–4.72)	4.1 (3.16–5.04)	6.8 (5.61–7.99)	1.9 (1.26–2.54)	7.2 (5.93–8.47)	19.2 (17.11–21.29)	9.3 (7.84–10.76)	5.2 (4.11–6.29)	2.3 (1.57–3.03)	5.8 (4.75–6.85)
Mansa, India (2017–2018)	2.3 (1.15–3.45)	2.3 (1.35–3.25)	4.2 (2.95–5.45)	1.2 (0.49–1.91)	4.3 (2.91–5.69)	16.6 (13.84–19.36)	10.2 (8.06–12.34)	4.8 (3.34–6.26)	1.5 (0.71–2.29)	4.5 (3.06–5.94)
Mumbai, India (2013–2017)	10.3 (9.95–10.65)	5.3 (5.05–5.55)	10.2 (9.83–10.57)	2.2 (2.03–2.37)	6.8 (5.50–7.10)	34.2 (33.54–34.86)	7.9 (7.58–8.22)	3.5 (3.29–3.71)	5.8 (5.52–6.08)	7.1 (6.80–7.40)
Delhi, India (2013–2017)	10.5 (10.07–10.93)	8.9 (8.50–9.30)	12.2 (11.68–12.72)	5.8 (5.46–6.14)	4.7 (4.40–5.00)	37.1 (36.27–37.93)	13.0 (12.50–13.50)	11.5 (11.03–11.97)	5.0 (4.68–5.32)	9.1 (8.69–9.51)
Kathmandu, Nepal (2019)	2.4 (1.46–3.34)	1.9 (1.05–2.75)	4.9 (3.49–6.31)	3.7 (2.51–4.89)	3.1 (1.99–4.21)	17.5 (15.09–19.91)	8.3 (6.61–9.99)	6.7 (5.12–8.28)	11.0 (8.99–13.01)	5.5 (4.16–6.84)
Bhutan (2019–2022)	1.9 (1.02–2.78)	1.8 (0.96–2.64)	2.6 (1.63–3.57)	2.4 (1.44–3.36)	7.0 (5.36–8.64)	9.9 (7.93–11.87)	19.5 (16.74–22.26)	5.5 (3.99–7.01)	7.7 (5.93–9.47)	6.3 (4.72–7.88)
Sri Lanka (2021)	13.9 (13.22–14.58)	8.3 (7.77–8.83)	11.6 (10.96–12.24)	1.0 (0.82–1.18)	4.1 (3.72–4.48)	40.5 (39.42–41.58)	9.1 (8.59–9.61)	0.9 (0.74–1.06)	4.0 (3.66–4.34)	9.9 (9.36–10.44)
England, United Kingdom (2013–2017)	2.3 (2.23–2.37)	3.4 (3.32–3.48)	75.6 (75.29–75.97)	1.3 (1.28–1.38)	6.1 (5.98–6.18)	92.5 (92.08–92.92)	7.7 (7.54–7.82)	1.5 (1.47–1.57)	27.5 (27.34–27.74)	9.5 (9.41–9.67)
United States (2013–2017)	1.8 (1.78–1.82)	4.2 (4.16–4.24)	73.7 (73.51–73.81)	1.7 (1.71–1.75)	9.4 (9.38–9.48)	90.7 (90.53–90.87)	6.0 (5.96–6.06)	1.7 (1.66–1.70)	32.1 (32.05–32.23)	7.9 (7.85–7.95)
Curitiba, Brazil (2013–2017)	2.7 (2.18–3.20)	3.1 (2.53–3.61)	43.7 (41.60–45.78)	1.5 (1.11–1.87)	6.3 (5.46–7.06)	52.6 (50.57–54.55)	11.1 (10.15–11.99)	1.8 (1.45–2.17)	10.9 (9.98–11.74)	5.8 (5.11–6.43)

95% Confidence interval in parenthesis * AAR: Age-adjusted incidence rate per 100,000 population

**Table 2.** Site-specific differences in the rural and urban regions of Muzaffarpur area (2018-2021).

ICD10	Site	Sex	Urban (AAR)	Rural (AAR)	Rate ratio (RR)	95% Confidence interval	
						Lower	Upper
**ALL**	**All Sites**	**Male**	**55.7**	**36.0**	1.6*	**1.36**	**1.76**
		**Female**	**60.7**	**43.0**	1.4*	**1.25**	**1.60**
C03-C06	Mouth	Male	11.5	4.5	2.5*	1.79	3.59
		Female	1.9	1.1	1.7	0.81	3.65
C01-C02	Tongue	Male	4.9	2.0	2.4*	1.44	4.03
		Female	2.0	0.7	2.8*	1.12	7.24
C23-C24	Gallbladder etc.	Male	2.3	1.7	1.3	0.69	2.45
		Female	6.3	4.9	1.3	0.88	1.87
C61	Prostate	Male	3.6	1.5	2.4*	1.32	4.2
C50	Breast	Female	19.0	8.9	2.1*	1.63	2.81
C53	Cervix uteri	Female	6.1	6.4	1.0	0.68	1.35
C56	Ovary	Female	3.4	1.1	3.1*	1.52	6.21

AAR: Age-adjusted incidence rate per 100,000 population

* Statistically significant at 95% CI

**Table 3.** Comparison of tobacco-related cancer rates in Muzaffarpur with other Indian cancer registries as well as those of the neighbouring and developed countries.

Cancer registry	Males		Females	
	Proportion to all site	AAR*	Proportion to all site	AAR*
Muzaffarpur, India (2018–2021)	35.2 (32.77–37.71)	14.2 (12.94–15.46)	11.3 (9.67–12.90)	5.5 (4.69–6.39)
Varanasi, India (2018–2019)	50.6 (48.63–52.49)	39.9 (37.67–42.19)	13.1 (11.58–14.37)	8.6 (7.51–9.71)
Sangrur, India (2017–2018)	35.4 (32.95–38.10)	26.5 (24.13–28.95)	14.6 (12.66–16.42)	11.8 (10.10–13.40)
Mansa, India (2017–2018)	31.9 (28.07–35.83)	18.7 (15.94–21.52)	15.9 (12.96–18.78)	10.7 (8.54–12.90)
Mumbai, India (2013–2017)	37.8 (37.33–38.36)	41.4 (40.64–42.08)	15.4 (15.01–15.76)	18.0 (17.51–18.47)
Delhi, India (2013–2017)	40.7 (40.17–41.24)	61.8 (60.75–62.93)	12.2 (11.86–12.61)	18.0 (17.42–18.62)
Kathmandu, Nepal (2019)	36.9 (33.73–39.91)	34.3 (30.65–38.03)	17.2 (15.03–19.54)	17.0 (14.48–19.48)
Bhutan (2019–2021)	23.0 (20.83–25.15)	31.9 (28.44–35.40)	13.9 (12.24–15.51)	24.5 (21.34–27.68)
Sri Lanka (2021)	43.3 (42.52–43.99)	66.4 (64.92–67.92)	12.7 (12.12–13.04)	18.2 (17.48–18.90)
England, United Kingdom (2013–2017)	18.5 (18.39–18.53)	77.4 (77.08–77.78)	13.1 (13.08–13.21)	41.6 (41.34–41.84)
United States (2013–2017)	25.7 (25.68–25.76)	77.9 (77.74–78.04)	16.8 (16.79–16.87)	42.3 (42.15–42.37)
Curitiba, Brazil (2013–2017)	18.7 (17.94–19.47)	47.0 (44.87–49.17)	8.6 (8.11–9.14)	17.5 (16.36–18.60)

*AAR: Age-adjusted incidence rate per 100,000 population, 95% Confidence interval in parenthesis

**Table 4.** Observed and relative survival among males and females of Muzaffarpur (2018).

Site (ICD-10)	Status			Observed survival (%)			Relative survival (%)		
	Alive	Death	Total	1 year	3 year	5 year	1 year	3 year	5 year
**Males**									
Mouth (C03-C06)	14	46	60	55.86 (42.59–67.21)	32.11 (20.76–44.00)	23.78 (13.94–35.09)	55.94 (42.64–67.30)	32.22 (20.83–44.14)	23.89 (13.99–35.26)
Prostate (C61)	6	16	22	73.27 (49.93–87.00)	40.55 (20.50–59.81)	26.64 (10.64–45.80)	73.46 (49.98–87.18)	40.79 (20.59–60.12)	26.89 (10.72–46.19)
Tongue (C01-C02)	7	15	22	54.31 (31.90–72.17)	35.75 (16.89–55.16)	31.39 (13.87–50.68)	54.35 (31.91–72.21)	35.79 (16.90–55.23)	31.46 (13.89–50.79)
**Females**									
Breast (C50)	29	66	95	76.92 (67.11–84.14)	42.97 (32.89–52.64)	32.58 (23.43–42.03)	77.01 (67.18–84.24)	43.11 (32.99–52.81)	32.75 (23.54–42.25)
Cervix uteri (C53)	10	47	57	57.29 (43.63–68.79)	31.99 (20.36–44.20)	21.44 (11.91–32.81)	57.37 (43.68–68.88)	32.09 (20.41–44.35)	21.56 (11.97–32.98)
Gallbladder (C23-C24)	0	40	40	14.62 (6.16–26.55)	0	0	14.64 (6.17–26.57)	0	0

95% Confidence interval in parenthesis

**Table 5.** Site-wise age-standardised relative survival rates of Muzaffarpur (2018).

Site (ICD-10)	Age-standardized relative survival (0–74 years) (%)			Age-standardized relative survival (all ages) (%)		
	1 year	3 year	5 year	1 year	3 year	5 year
**Male**						
Mouth (C03-C06)	54.60 (40.85–66.43)	32.83 (21.17–44.98)	25.59 (15.18–37.32)	55.08 (41.90–66.44)	31.79 (20.73–43.40)	23.47 (13.93–34.45)
Prostate (C61)	75.16 (50.50–88.75)	45.32 (23.28–65.06)	30.41 (12.68–50.36)	72.92 (49.75–86.69)	41.22 (21.09–60.37)	27.67 (11.47–46.66)
Tongue (C01-C02)*	54.58 (33.86–71.27)	36.42 (18.33 - 54.81)	31.90 (14.72 - 50.59)	54.58 (33.86 - 71.27)	36.42 (18.33 - 54.81)	31.90 (14.72–50.59)
**Female**						
Breast (C50)	76.74 (66.89–84.00)	43.46 (33.12–53.35)	32.39 (23.12–41.98)	76.93 (67.37–84.02)	43.32 (33.25–52.97)	32.84 (23.71–42.26)
Cervix uteri (C53)	58.57 (44.28–70.38)	34.07 (22.03–46.48)	20.73 (11.12 –32.39)	56.23 (42.42–67.94)	31.69 (20.51–43.44)	19.28 (10.36–30.27)
Gallbladder (C23-C24)	15.40 (06.52–27.74)	0.00	0.00	15.01 (06.37–27.08)	0.00	0.00

* There were no tongue cancer cases above 74 years of age
